# Jie-du granule preparation for the treatment of advanced hepatocellular carcinoma: a retrospective cohort study of 177 patients

**DOI:** 10.18632/oncotarget.12887

**Published:** 2016-10-25

**Authors:** L.Y. Chen, X.F. Zhai, Z. Chen, J.F. Zhu, P.A. Qian, H.T. Zhao, C.Q. Ling

**Affiliations:** ^1^ Department of Traditional Chinese Medicine, Changhai Hospital, Second Military Medical University, Shanghai, China; ^2^ Department of Liver Disease, Shanghai Municipal Hospital of Traditional Chinese Medicine, Shanghai, China

**Keywords:** hepatocellular carcinoma, Jie-du granule, best supportive treatment, retrospective cohort study

## Abstract

**Objective:**

To compare the clinical efficacy of Jie-du granule preparation versus best supportive treatment in patients with advanced hepatocellular carcinoma.

**Methods:**

A retrospective cohort study was carried out in patients with advanced liver cancer. Patients were divided into Jie-du granule treatment (JD) and best supportive treatment (BST) groups. The main outcomes included median overall survival time.

**Results:**

A total of 177 patients with Barcelona Clinic Liver Cancer stage C receiving JD granule treatment or BST were enrolled between January 2012 and December 2014. The overall median survival time was 6.2 months (95% confidence interval [CI] 4.546-7.854) in the JD group versus 4 months (95% CI 3.471-4.529) in the BST group. Significant independent risk factors were alpha-fetoprotein (*P* = 0.048), Child-Pugh class (*P =* 0.005), vascular invasion (*P* = 0.003), and extrahepatic metastasis (*P* = 0.0018). For patients with two or fewer of these independent risk factors, the overall median survival of those treated with JD was significantly longer than that of patients receiving BST (*P* < 0.05).

**Conclusion:**

Jie-du granule preparation may prolong survival of patients with advanced HCC.

## INTRODUCTION

Many patients with hepatocellular carcinoma (HCC) in China are diagnosed with Barcelona Clinic Liver Cancer stage C (BCLC-C) disease. Sorafenib and best supportive treatment (BST) have been recommended as standard treatments for HCC patients staged as BCLC-C [1]. However, sorafenib is not widely used in clinical practice in China because it is expensive. Instead, many HCC patients with advanced cancer stage and/or poor liver functional reserve receive traditional Chinese medicine (TCM) treatment. Jie-du granule preparation is a commonly used Chinese herbal medicine formula for HCC [2–3]. Under the supervision of Professor CQ Ling and Professor JF Zhu, more than 20,000 HCC patients have received JD granule treatment from 1996 to 2016. However, few studies have focused on the therapeutic efficacy of Jie-du granule for BCLC-C HCC, raising the question of whether treatment with Jie-du granule alone might be superior to best supportive treatment. Therefore, we conducted a retrospective cohort study to (i) compare the efficacy of Jie-du granule and best supportive treatment, and (ii) evaluate whether TCM alone is an effective treatment approach for advanced HCC.

## MATERIALS AND METHODS

### Study population

A consecutive case series of HCC patients treated with Jie-du granule or best supportive treatment at Changhai Hospital and Shanghai Traditional Chinese Medicine Hospital from January 2012 to December 2014 was studied retrospectively. The inclusion criteria for the study population were as follows: (i) HCC diagnosed according to the criteria issued by the European Association for the Study of Liver Disease/American Association for the Study of Liver Disease [4]; (ii) confirmed BCLC-C; (iii) patients received only traditional Chinese treatment, of which Jie-du granule preparation was dominant (only received treatment with oral JD guanule preparation), or only best supportive treatment. The exclusion criteria were as follows: (i) severe disease or dysfunction of heart, brain, or kidney; (ii) receiving other anticancer treatment after enrollment; (iii) second primary malignancy. The enrolled patients were divided into the JD (Jie-du granule) group and the BST (best supportive treatment) group depending on the treatment received. The study protocol was approved by the ethics committees of all participating hospitals. Collection of informed consent was waived given the retrospective nature of the study.

### Medical treatment

All patients in the JD group received only Jie-du granule preparation (produced by the Good Manufacturing Practice-certified Tianjiang Pharmaceutical Factory, Jiangsu, China; Production License No. Su ZzY20010266), at an oral dose of 8 g (equal to 80 g of raw herbal material) twice a day, 30 minutes after a meal. The JD granule preparation is composed of four traditional Chinese herbal medicines, root of *Actinidia valvata*, root of *Salvia chinensis*, bulb of *Cremastra appendiculata*, and gizzard membrane of *Gallus gallus domesticus*, which are extracted by hot water and lyophilization and are present in a proportion of 1:1:0.4:0.4 [4]. All patients in the BST group received best supportive therapies selected according to the preferred practices at each center.

### Follow-up evaluation

The patients were followed up by telephone and the time of death was recorded. The end of the follow-up period was either death or May 2015. The primary endpoint was overall survival (OS), which was measured from the time of initial treatment in the study until the time of death or May 2015.

### Statistical analysis

Survival was assessed according to the Kaplan-Meier method, and curves were compared using the log-rank test. The Cox proportional hazard model was used to assess the prognostic values of the variables. The following parameters were selected: age, gender, etiology, α-fetoprotein (AFP), extrahepatic metastasis, lymphatic metastasis, vascular invasion, tumor size, and Child-Pugh classification. Statistical analyses were carried out using SPSS software, version 20.0 (SPSS, Inc., Chicago, IL).

## RESULTS

### Patient characteristics

A total of 194 BCLC-C stage HCC patients were enrolled from January 2012 to December 2014. We excluded 17 patients whose family members did not cooperate in the follow-up, therefore 177 patients who met the inclusion criteria were finally evaluated. Of these patients, 79 were in the JD group and 98 in the BST group. Most patients in the JD group only received long-term maintenance treatment with oral JD granule preparation. Only 3 patients received other herbal medicine treatment (no more than 4 kinds of herbals) because of fatigue, abdominal distension and other symptoms. The two groups were comparable in age, sex, Child-Pugh, disease burden, etiology, and AFP. Detailed baseline clinical characteristics of these 177 patients are summarized in Table 1.

**Table 1:** Patient baseline and clinical characteristics (*n* = 177)

		JD	BST	P
Age, y		58.4±12	60.3±14.5	0.34
Sex	Male	67	78	
	Female	12	20	0.37
Child-Pugh	A	32	26	
	B	47	71	0.05
Etiology				
HBV-Ag	-	12	13	
	+	67	84	0.73
Disease burden				
Lymphatic metastasis	-	36	53	
	+	43	45	0.53
Vascular invasion	-	33	31	
	+	46	67	0.16
Main branch		17	10	
Left branch		21	10	
Right branch		16	18	
Other		3	8	0.11
Extrahepatic metastasis	-	52	53	
	+	27	45	0.07
Tumor size	<5cm	37	53	
	≥5cm	42	45	0.55
Tumor number	Single	8	10	
	Multiple	59	67	
	Diffuse	4	12	0.25
Tumor location	Left lobe	9	49	
	Right lobe	23	10	
	Diffuse	12	18	0.41

### Survival of patients

**Figure 1:**
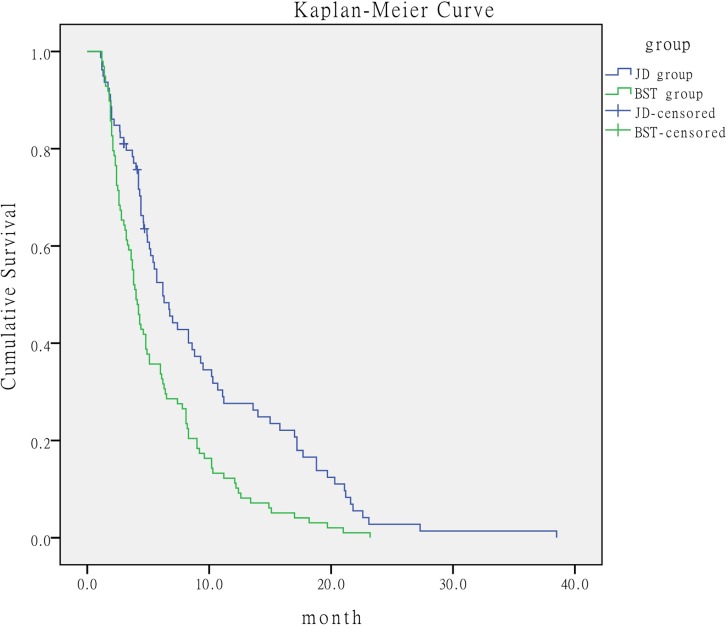
Comparison of survival time of patients according to treatment

Of the 79 patients in the JD group, 5 patients survived and the remaining 74 patients died. All 98 patients in the BST group died. The median OS time in the JD group was 6.2 months (95% confidence interval [CI], 4.379-8.221) compared with 4 months (95% CI, 3.471-4.529) in the BST group. The log-rank analysis showed that the median survival time in the JD group was significantly longer than that in the BST group (*P* < 0.001) (Figure 1).

### Prognostic analysis

In the multivariate Cox model, the following four parameters were considered as risk factors: Child-Pugh B class, AFP ≥400 ng/dL, vascular invasion, and extrahepatic metastasis, with corresponding hazard ratios (HR) of 1.669 (95% CI 1.172-2.378), 1.385 (95% CI 1.003-1.913), 1.706 (95% CI 1.201-2.423), and 1.516 (95% CI 1.075-2.137) respectively. These variables were independent prognostic risk factors (Table 2).

**Table 2:** Predictors of overall survival in 177 HCC patients

Variable	Univariate analysis			Multivariate analysis		
	HR	95%CI	*P*	HR	95%CI	*P*
Child-Pugh(B/A)	1.698	1.223-2.358	0.002	1.669	1.172-2.378	0.005
AFP(≥400 /<400 ng /dl)	1.264	0.934-1.709	0.129	1.385	1.003-1.913	0.048
Vascular invasion(yes/no)	1.471	1.074-2.017	0.016	1.706	1.201-2.423	0.003
Extrahepatic metastasis(yes/no)	1.370	1.009-1.860	0.044	1.516	1.075-2.137	0.018
Treatment(JD /BST)	1.791	1.310-2.448	0.000	1.399	1.001-1.956	0.050
Gender(female /male)	0.977	0.663-1.440	0.907			
Age	0.987	0.977-0.998	0.022			
Lymphatic metastasis (yes/no)	1.024	0.758-1.385	0.875			
HBV-Ag(+/−)	0.860	0.564-1.312	0.484			
Tumor size(≥10/<10cm)	1.010	0.741-1.378	0.949			

### BCLC-C stage subgroups

**Figure 2:**
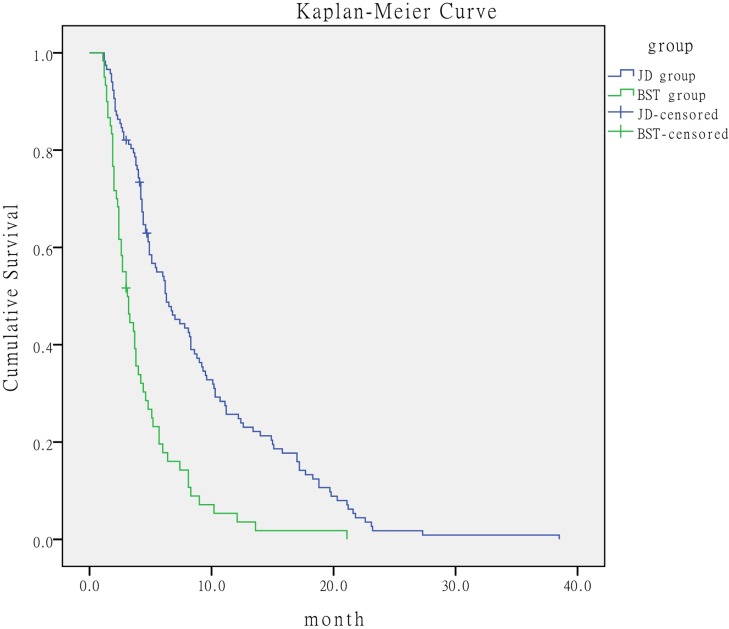
Comparison of survival time of patients with ≤2 prognostic risk factors according to treatment

**Figure 3:**
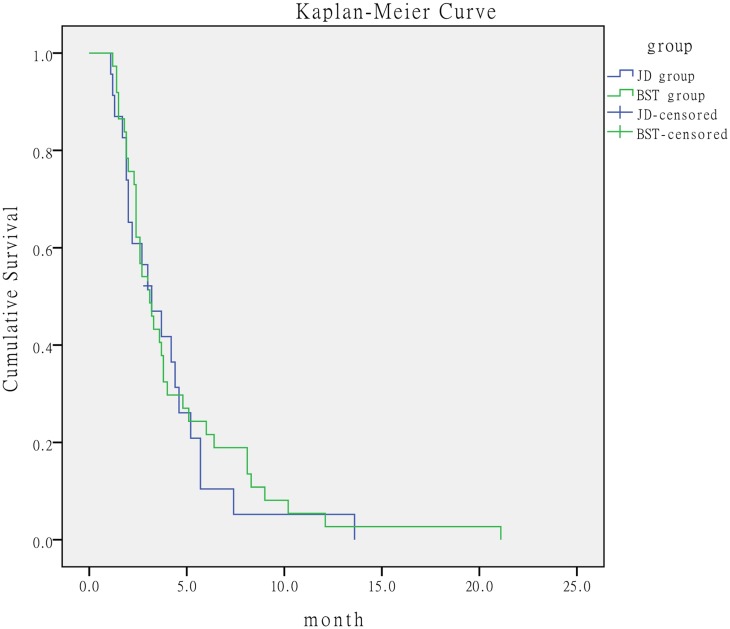
Comparison of survival time of patients with ≥3 prognostic risk factors according to treatment

We subdivided the BCLC-C stage into four subgroups according to the presence of independent prognostic risk factors of Child-Pugh, AFP, vascular invasion, and extrahepatic metastasis as follows: no or 1 prognostic risk factor, 2 prognostic risk factors, 3 prognostic risk factors, and 4 prognostic risk factors. Patients with 0-2 prognostic risk factors in the JD group had better outcome than the corresponding patients in the BST group. The median survival time was 8.8 months (95% CI, 5.570-12.030) in the JD group *versus* 4.6 months (95% CI, 3.835-5.365) in the BST group. The log-rank test showed that the median survival for the JD group was significantly longer than that for the BST group (*P* < 0.001) (Figure 2). When the number of prognostic risk factors ranged from 3 to 4, the median survival time was 3.2 months (95% CI, 1.794-4.606) in the JD group *versus* 3.1 months (95% CI, 2.266-3.934) in the BST group, with no significant difference between the two groups (*P* = 0.659) (Figure 3).

## DISCUSSION

Sorafenib and supportive treatment are recommended as standard treatments for HCC by the American Association for the Study of Liver Diseases (ASSLD). In the clinical trial of sorafenib in the Asia-Pacific region (ORIENTAL), median OS in the sorafenib group was 6.5 months compared with 4.2 months in the placebo group [5]. In our study, the median OS time was 6.2 months (95% CI, 4.379-8.221) in the JD group *versus* 4 months (95% CI, 3.471-4.529) in the BST group, which was similar to the results of the ORIENTAL study. This result suggested that the efficacy of Jie-du granule preparation might be similar to that of sorafenib. Both treatments could prolong survival of patients with advanced HCC and both showed better efficacy than supportive treatment. However, the price of the Chinese herb is much lower than that of sorafenib.

The patients were divided into four subgroups based on 4 independent prognostic risk factors (Child-Pugh, AFP, vascular invasion, and extrahepatic metastasis). The comparison of survival time of patients in different subgroups demonstrated the heterogeneity of BCLC-C HCC patients, and further showed that patients with vascular invasion, extrahepatic metastasis, or poor liver function had higher mortality than those without these prognostic risk factors. Therefore, a single therapy mode for all patients might not be appropriate. We conclude that patients with two or fewer of these prognostic risk factors should receive traditional Chinese medicine treatment rather than best supportive treatment. Moreover, our data strongly suggest that stratification of BCLC-C is of great significance for optimal treatment of advanced HCC.

Jie-du granule preparation is composed of *Salvia chinensis* Benth, root of *Actinidia valvata*, bulb of *Cremastra appendiculata*, and *ventriculi galli mucosa* [6]. Previous trials have demonstrated that these cancer-fighting Chinese herbs inhibited development of liver cancer [2, 3, 7, 8]. Shim and Xin proved that *Salvia chinensis* Benth and bulb of *Cremastra appendiculata* showed effective antiangiogenesis and cytotoxic activity *in vitro* against the BEL-7402 and SMMC-7721 tumor cell lines [9]. Zhang demonstrated that the active fraction from *Actinidia valvata* had an inhibitory effect on transplanted H22 mouse tumor cells [10]. The underlying mechanism may be through modulation of the cell cycle and induction of cellular apoptosis.

The results of our study demonstrated that the traditional Chinese medicine prolonged survival of patients with advanced HCC. Moreover, the median survival time of patients who received Jie-du granule was similar to that of patients who received sorafenib, suggesting that the efficacy of Jie-du granule is probably similar to that of sorafenib. A randomized prospective trial has been conducted to compare the efficacy of Jie-du granule and sorafenib. The results of this trial will be revealed at the end of 2016.
